# Testing the Accuracy of the ARIMA Models in Forecasting the Spreading of COVID-19 and the Associated Mortality Rate

**DOI:** 10.3390/medicina56110566

**Published:** 2020-10-27

**Authors:** Ovidiu-Dumitru Ilie, Alin Ciobica, Bogdan Doroftei

**Affiliations:** 1 Department of Research, Faculty of Biology, “Alexandru Ioan Cuza” University, Carol I Avenue, no 11, 700505 Iasi, Romania; 2 Faculty of Medicine, University of Medicine and Pharmacy “Grigore T. Popa”, University Street, no 16, 700115 Iasi, Romania; bogdandoroftei@gmail.com

**Keywords:** SARS-CoV-2, COVID-19, epidemiology, incidence, prevalence, mortality rate, forecasting

## Abstract

*Background and objectives:* The current pandemic of SARS-CoV-2 has not only changed, but also affected the lives of tens of millions of people around the world in these last nine to ten months. Although the situation is stable to some extent within the developed countries, approximately one million have already died as a consequence of the unique symptomatology that these people displayed. Thus, the need to develop an effective strategy for monitoring, restricting, but especially for predicting the evolution of COVID-19 is urgent, especially in middle-class countries such as Romania. *Material and Methods:* Therefore, autoregressive integrated moving average (ARIMA) models have been created, aiming to predict the epidemiological course of COVID-19 in Romania by using two statistical software (STATGRAPHICS Centurion (v.18.1.13) and IBM SPSS (v.20.0.0)). To increase the accuracy, we collected data between the established interval (1 March, 31 August) from the official website of the Romanian Government and the World Health Organization. *Results:* Several ARIMA models were generated from which ARIMA (1,2,1), ARIMA (3,2,2), ARIMA (3,1,3), ARIMA (3,2,2), ARIMA (3,1,3), ARIMA (2,2,2) and ARIMA (1,2,1) were considered the best models. For this, we took into account the lowest value of mean absolute percentage error (MAPE) for March, April, May, June, July, and August (*MAPE_March_* = 9.3225, *MAPE_April_* = 0.975287, *MAPE_May_* = 0.227675, *MAPE_June_* = 0.161412, *MAPE_July_* = 0.243285, *MAPE_August_* = 0.163873, *MAPE_March – August_* = 2.29175 for STATGRAPHICS Centurion (v.18.1.13) and *MAPE_March_* = 57.505, *MAPE_April_* = 1.152, *MAPE_May_* = 0.259, *MAPE_June_* = 0.185, *MAPE_July_* = 0.307, *MAPE_August_* = 0.194, and *MAPE_March – August_* = 6.013 for IBM SPSS (v.20.0.0) respectively. *Conclusions:* This study demonstrates that ARIMA is a useful statistical model for making predictions and provides an idea of the epidemiological status of the country of interest.

## 1. Introduction

Towards the end of 2019, local hospitals from the Hubei region, Wuhan city begun to report by the day more and more cases of severe pneumonia with an unknown etiology. It was difficult for clinicians to establish a diagnosis on the basis of the unique symptomatology that the first patient had. Fortunately, in a relatively short interval it was revealed that the so-called patient zero was infected with a novel beta-coronavirus. Already known as severe acute respiratory coronavirus 2 (SARS-CoV-2) after its successor, it was demonstrated that person-to-person transmission ultimately causes the coronavirus disease (COVID-19) [1,2,3].

Intriguingly, 2019-nCoV is a zoonotic family member, and for a long time it was speculated that *Rhinolophus sinicus* is the natural host of SARS-CoV. Unfortunately, no clear evidence was found that incriminates the horseshoe bat, all remaining at a hypothetical stage even after almost a year since the first case was reported. Most likely, these assumptions were made based on the current knowledge that the bat is a natural reservoir for pathogens. Because of its novelty, more than fifty people were confirmed as SARS-CoV-2-infected patients by the beginning of 2020 [4].

In retrospect, humanity has never faced such a crisis since the Spanish flu between 1918 and 1919/1920, with figures indicating that it caused the death of fifty-one hundred million people [5]. Even if both clinicians and researchers are in a timed battle against this virus, the latest statistics issued by the World Health Organization (WHO) suggest that over twenty million people are positive, and approximately eight hundred thousand have died despite their best efforts (https://covid19.who.int/).

Considering the uncontrolled and fulminant spreading of SARS-CoV-2, concomitantly with its identification it was demonstrated that the elderly and those who have associated chronic diseases are the most predisposed [6]. However, these figures vary, not because of the lack of data, but rather the finite capacities in the epidemiological surveillance. Based on the aforementioned, the need for a reliable and efficient strategy for planning health infrastructure is all more imperative, especially for mid-class countries. Compared with Westernized countries that have all the resources necessary, in Romania, on the other hand, the situation may reach the critical point and soon be cataloged as the second Lombardia.

There is an increasing trend in the current literature regarding the possible epidemiological course of COVID-19. Both mathematical and statistical models are crucial to determine short and long case estimates [6]. One example is represented by the AutoRegressive Integrated Moving Average (ARIMA) model that has been successfully applied in the past to estimate the prevalence and incidence of numerous other highly infectious diseases (Table 1) [7].

Unlike the other studies conducted, the present study aims to estimate COVID-19 cases through ARIMA using two distinct statistical software (IBM SPSS and STATGRAPHICS) in order to test their reliability and accuracy. It also aims to present the evolution of the mortality rate in Romania considering the high, almost double reports between the number of positive cases/deaths in the last thirty days compared to the same intervals of the previous months.

## 2. Material and Methods

### 2.1. Data

The daily prevalence data of COVID-19 was taken from The Ministry of Internal Affairs of Romania (https://www.mai.gov.ro), and compared to the figures reported by the World Health Organization (WHO) (https://covid19.who.int/). An MS Excel was used to build a time-series database.

Even though the first case in Romania was reported back on 27 February, we decided the following: (1) in order to test the accuracy of the ARIMA models, the established interval was divided into small (1 month) subdivisions with fourteen days forecast of the next month and comparing the numbers reported daily by the Romanian Government and WHO; (2) to perform a forecast from the so-called point zero (27 February) until the present day, 31 August, with also a fourteen days forecast.

Descriptive statistics of the COVID-19 data for the established intervals (1 March–31 March, 1 April–30 April; 1 May–31 May; 1 June–30 June; 1 July–31 July, 1 August–31 August, and 27 February–31 August) are given in Table 2. The current situation in Romania (31 August) is as follows: 85,833 confirmed cases and 3539 deaths. At least thirty observations are recommended for an optimum ARIMA model [15].

Thus, the data set was used to conduct and analyze a case estimation model starting from the assumption according to which it will be useful in the future to predict the evolution of COVID-19 in Romania. Therefore, a time-series containing at least 45 data was used to predict SARS-CoV-2 prevalence in Romania over the next two weeks with a 95% confidence interval (CI).

Initially, the outbreak did not affect Romania significantly, but starting from 23 July, the number of positive cases exceeded 1000. Since then, only on 4, 11, 18, 24, 25 August were registered <1000 cases per day, the highest number being reported on 28 August with 1504 confirmed cases and 38 deaths.

### 2.2. The ARIMA Model

A time-series, as the name suggests, is just a succession of data points indexed in a time order [16] dedicated to generating statistical data. More precisely, are used to perform predictions of values of a series [17], ARIMA becoming a simple-to-use algorithm since it was introduced in the 1970s [15]. ARIMA is preferred to the detriment of other models due to the fact it takes into account all (in)dependent variances. Nevertheless, beyond fitting for a large sphere of data, through seasonality to cyclicity a temporal dependency can be modeled.

In summary, autoregressive integrated moving average (ARIMA) technique is used for tracking linear tendencies, the entire concept constituting a mixture or being denoted by three orderly parameters. Non-seasonal ARIMA’s parameters AR(*p*) (auto regression) represents the order of autoregression, MA(*q*) (moving average) the order of moving average, whereas I(*d*) is the degree of difference.

Viewed or described as a time-series, *Y*t represents a succession of independent arguments on the basis of a time *t* [18]. A deterministic/stochastic time-series could be explained by the following function, *Y*t = *f/X*(*t*), where *X* is just a random variable. Thus, AR(*p*) (Equations (1a) and (1b)) predict the future value based on previous *p*-time observations as inputs, *θ* or *Φ* is the multiplying coefficient, ε_t_ or ω is the random error or white noise at a time *t* and *µ*, the mean of a series. In cases of a stationary time-series, the average of the ε_t_ or ω_t_ is 0, the variance being noted as σ^2^:

(1a) $$Y_{t}\;=\;\alpha +\Phi_{1}Y_{t-1}+\;\Phi_{2}Y_{t-2}+\cdots +\;\Phi_{p}Y_{t-p}+\;\varepsilon_{t}\;,$$

or:

(1b) $$Y_{t}\;=\;µ+\overset{p}{\underset{i=1}{\sum }}\left(\Phi_{i}Y_{t-i}\right)+\;\omega_{t}\;.$$

Here, δ or α have the same value = constant. The polynomial’s MA(*q*) (Equations (2a) and (2b) time-series as a *q*^th^ degree can be found such as follows:

(2a) $$Y_{t}\;=\;µ+\varepsilon_{t}+\;\theta_{1}\;\varepsilon_{t-1}+\;\theta_{2}\;\varepsilon_{t-2}+\cdots +\;\theta_{q}\;\varepsilon_{t-q}\;,$$

or:

(2b) $$Y_{t}\;=\;µ+\overset{q}{\underset{j=1}{\sum }}\left(\theta_{j}\omega_{t-1}\right)+\;\omega_{t}\;.$$

Therefore, AR(*p*)MA(*q*)’s expression is obtained by combining *p* and *q*, mathematically being represented in Equations (3a) and (3b) [19]:

(3a) $$Y_{t}\;=\;\delta +\;\Phi_{1}Y_{t-1}+\cdots +\Phi_{p}Y_{t-p}+\;\varepsilon_{t}+\;\theta_{1}\;\varepsilon_{t-1}+\cdots +\;\theta_{q}\;\varepsilon_{t-q}\;,$$

or:

(3b) $$Y_{t}\;=\;µ+\overset{p}{\underset{i=1}{\sum }}\left(\Phi_{i}Y_{t-i}\right)+\overset{q}{\underset{j=1}{\sum }}(\theta_{j}\omega_{t-j})+\;\omega_{t}\;.$$

On the other hand, there are also circumstances when the time-series is not stationary. In such cases, it should be verified if this condition is satisfied or not; if not, it can be made stationary by adding another variable *d*. Once ∆Y “take over” Yt’s non-stationary differences, ∆Y can be explained as follows: (Equation (4)), with *L* representing the likelihood of the data:

(4) $$\Delta Y_{t}\;=\;Y_{t}-Y_{t-1}=Y_{t}-LY_{t}=\;Y_{t}^{\prime }\;.$$

For testing the accuracy of our model, we analyzed the performance of three factors known under the name of root mean square error (RMSE), mean absolute error (MAE), and mean absolute percentage error (MAPE) (Equations (5)–(7)):

(5) $$\mathrm{MAE}=\frac{1}{n}\overset{n}{\underset{i=1}{\sum }}\left|Y_{i}-\;\hat{Y_{i}}\right|\;,$$

(6) $$\mathrm{MAPE}=\frac{100}{n}\;x\overset{n}{\underset{i=1}{\sum }}\left|\frac{Y_{i\;}-\;\hat{Y_{i}}}{Y_{i}}\right|\;,$$

(7) RMSE= ∑i=1n(Yi ^−Yi)^2n .

For congruity, MAE, MAPE, and MRSE’s values must be low, all analyses being performed using STATGRAPHICS Centurion (v.18.1.13) and IBM SPSS (v.20.0.0) software with statistically significant levels of *p* < 0.05.

### 2.3. Mortality Rate

We collected data aiming to determine the mortality rate depending on the sex of each individual, the median age ranging between <10 as the minimum and >80 as the maximum limit of people who have died, who had associated comorbidities, and were hospitalized in intensive care units (ICUs) between the established intervals. All these parameters were calculated using Excel software. Unfortunately, there are some limitations in this context. More specifically, the figures related to the sex of patients, the median age, the associated comorbidities, and at ICU are incomplete as a consequence of lack of management from the Romanian government during this pandemic. Based on the aforementioned, we were able to collect data from the last several months; 11 June for sex, median age, and associated comorbidities, and 17 March for ICU patients.

## 3. Results

**Figure 1:**
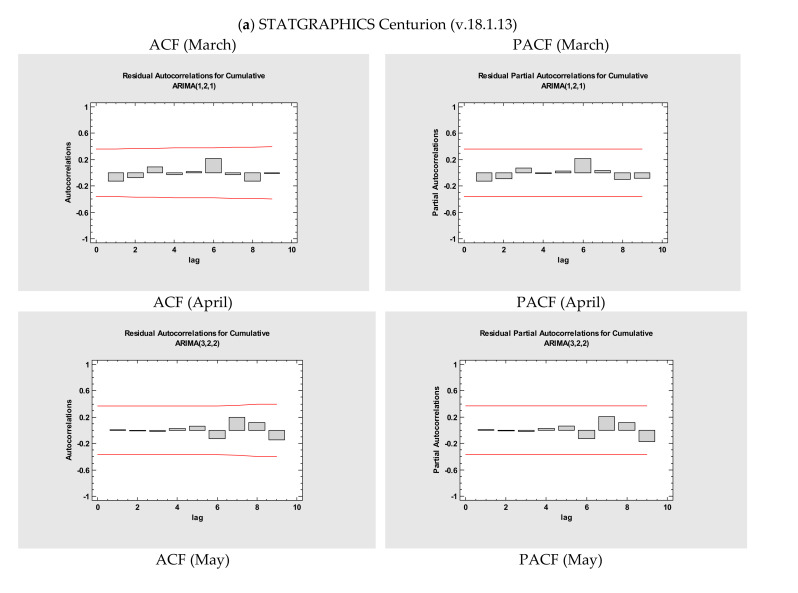

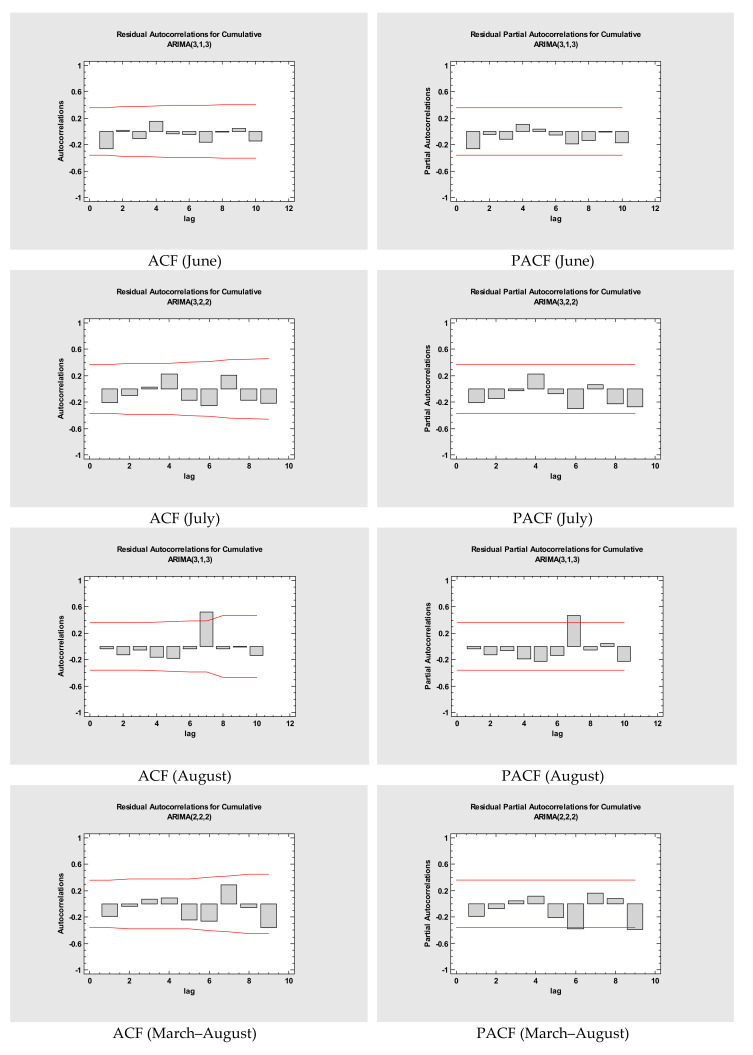

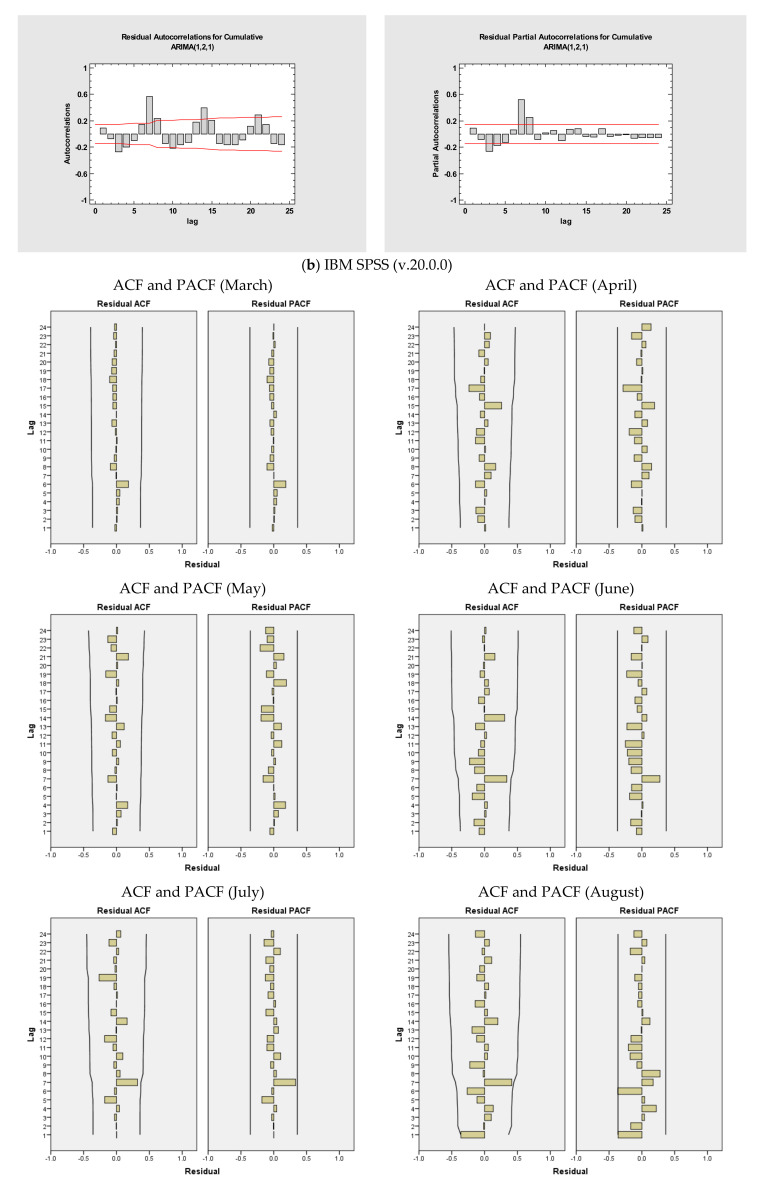

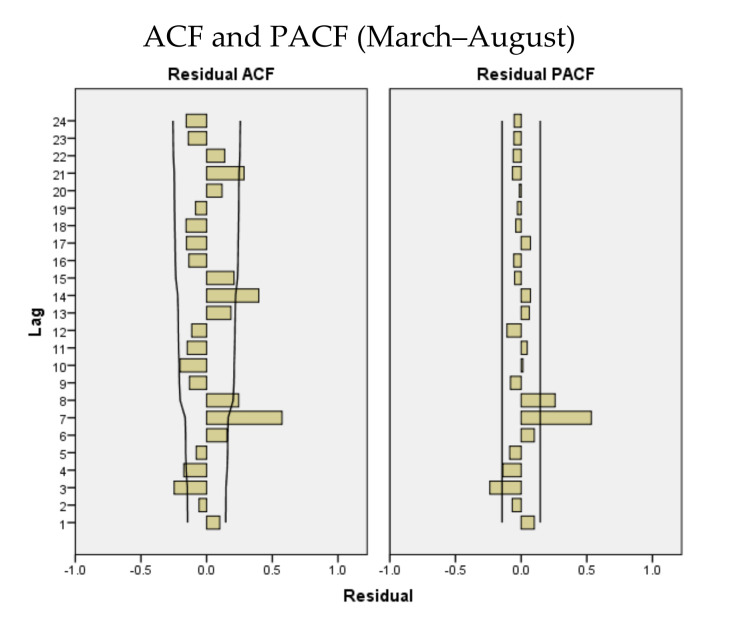
The estimated AutoCorrelation Function (ACF) and Partial AutoCorrelation Function (PACF) graphs to predict the epidemiological trend of COVID-19 in Romania performed by using (**a**) STATGRAPHICS Centurion (v.18.1.13) and (**b**) IBM SPSS (v.20.0.0).

Building an ARIMA model for any given time-series involves the checking of four steps: assessment of the model, estimation of parameters, diagnostic checking, and prediction. The first, which is otherwise imperative, is to verify if the mean, variance, and autocorrelation of the time-series are consistent throughout the established interval [20]. Therefore, two-time-series plots, autocorrelation function (ACF), and partial autocorrelation function (PACF) (Figure 1) graphs were generated to test the seasonality and stationarity. ACF is a statistical metric that determines whether the prior values are related to the latest values of not, while PACF the value of the correlation coefficient between its time lag and the variable [13]. Both are imperative in detecting misspecification, the model performance being measured by Akaike information criteria expression, and the Bayesian information criterion of Schwarz (BIC) [21]. Estimated autocorrelations for Romania are presented in Figure 1; the straight lines indicate the limit of two standard deviations and the bars that extend beyond the lines suggest statistically meaningful autocorrelations.

Additionally, a series of ARIMA models were created, and their performances were compared using various statistical tools. All statistical procedures were performed on the transformed COVID -19 data. ARIMA models with the lowest MAPE values were considered the most optimum model. Among the tested models, ARIMA (1,2,1), ARIMA (3,2,2), ARIMA (3,1,3), ARIMA (3,2,2), ARIMA (3,1,3), ARIMA (2,2,2), ARIMA (1,2,1) were chosen as the best models for Romania. The models where COVID-19 data fitted are presented in Figure 1 and Table 3 and Table 4 with a minimum *MAPE_March_* = 9.3225, *MAPE_April_* = 0.975287, *MAPE_May_* = 0.227675, *MAPE_June_* = 0.161412, *MAPE_July_* = 0.243285, *MAPE_August_* = 0.163873, *MAPE_March – August_* = 2.29175, *MAPE_March_* = 57.505, *MAPE_April_* = 1.152, *MAPE_May_* = 0.259, *MAPE_June_* = 0.185, *MAPE_July_* = 0.307, *MAPE_August_* = 0.194, and *MAPE_March – August_* = 6.013, respectively.

**Figure 2:**
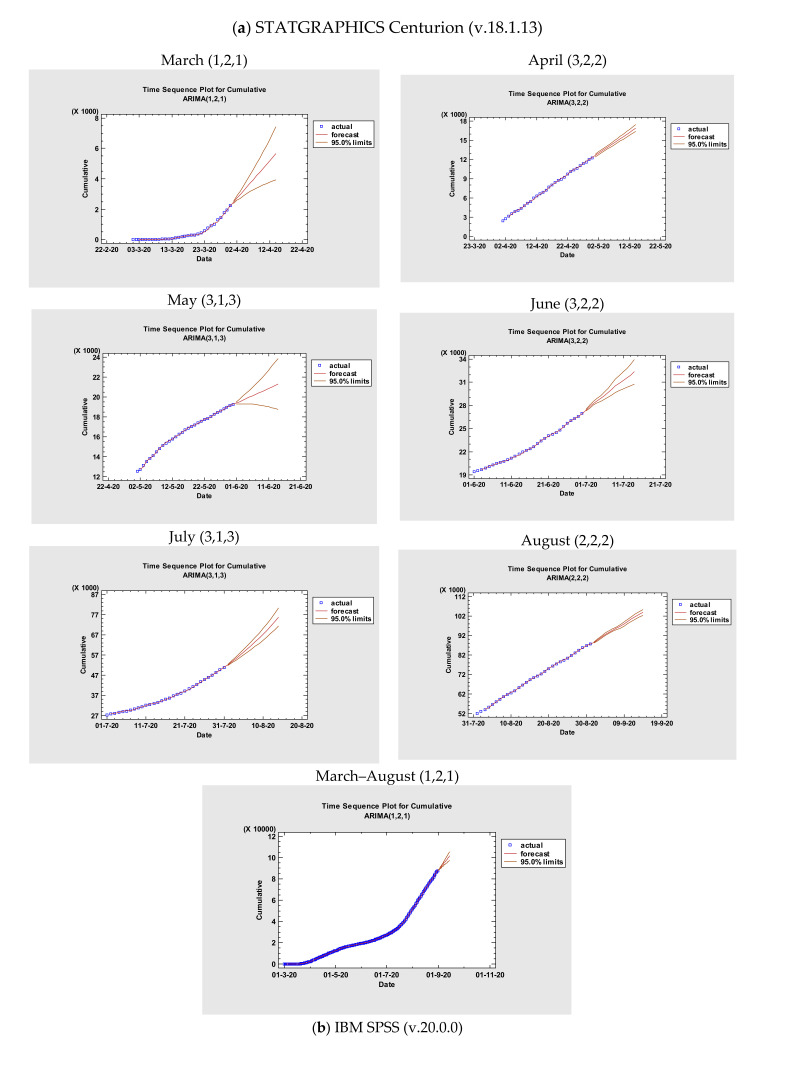

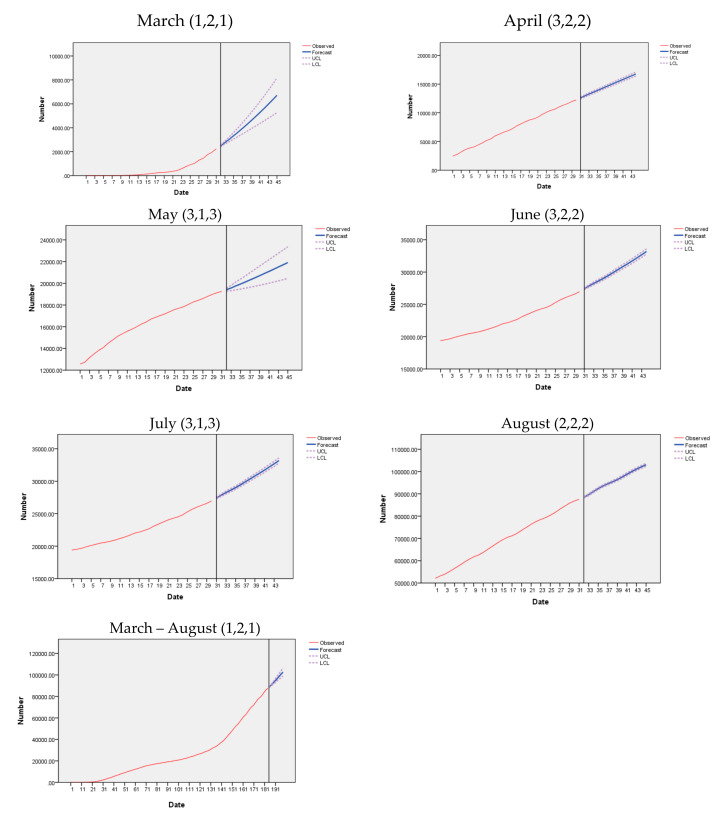
Time-series plots for the best ARIMA models through (**a**) STATGRAPHICS Centurion (v.18.1.13) and (**b**) IBM SPSS (v.20.0.0).

In Table 4, the parameter estimates for the best models are presented. The fitted and predicted values are presented in Figure 2. As seen in Table 5 for both software, the next two weeks estimate of confirmed cases may be between 2450.74–5673.29, 12,616.5–16,896.3, 19,400.9–21,280.5, 27,404.9–32,340.9, 52,247.6–75,717.2, 88,483.4–103,777, 88,427.4–101,440, respectively through STATGRAPHICS Centurion (v.18.1.13). For IBM SPSS (v.20.0.0), the forecast for the next two weeks is as follows: 2478.78–6715.00, 12,599.76–16,756.08, 19,412.18–21,910.55, 27,405.69–33,181.42, 52,168.29–67,467.42, 88,444.38–103,059.41, and 88,451.83–102,656.81 for March, April, May, June, July, August, and March–August.

**Figure 3:**
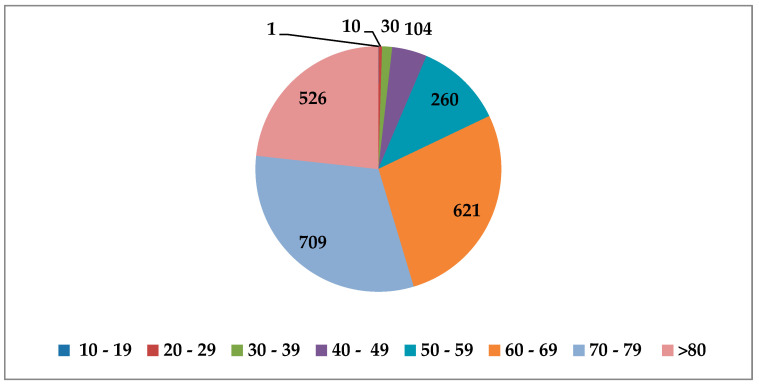
The number of deaths depending on the age group.

**Figure 4:**
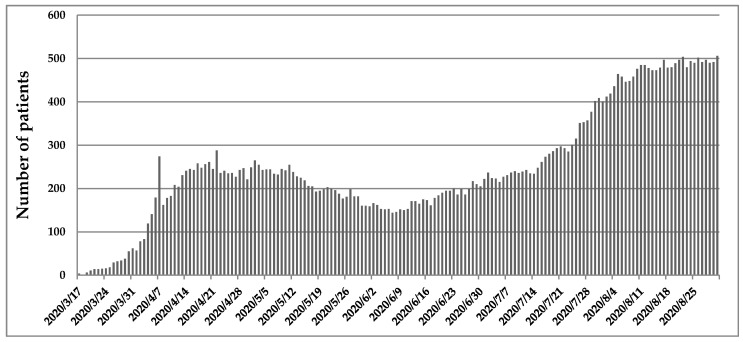
The total number of patients hospitalized in ICU.

Regarding the mortality rate, since 11 June until 31 August a total of 2261 patients were identified, from which 1356 (59.97%) were male and 905 (40.02%) were female. The most affected age group were people aged between 70 and 79 years, where SARS-CoV-2 caused the death of 709 people, followed by people between 60 and 69 years with 621 deaths and >80 with 526 deaths (Figure 3). On the other hand, a total of 405 people died, from which 260 had between 50 and 59 years, 104 between 40 and 49 years, 30 between 30 and 39 years, 10 between 20 and 29 years, 1 between 10 and 19 years, and 0 with less than 10 years old. From the total number of 2261 people, 2184 had comorbidities (96.6823%), and 77 not. As well, since 17 March when the first 4 people were confirmed, the total number registered until 31 August was 506 (Figure 4).

## 4. Discussion

Based on our results, it can be concluded that Romania will face an even higher number of infections which can exceed one hundred thousand. In terms of the number of deaths, these figures are not comparable with other countries such as Italy, Spain, or France. The probability of exceeding 1000 is very small, especially due to the high longevity rate of people from other states compared to Romania.

According to the current literature, this is the first study of such a manner. Thus, the idea of testing the accuracy of the ARIMA model using two distinct statistical software is novel, all the more so as middle-class countries do not have the resources necessary or a reliable strategy in restraining the rate of contagion or transmissibility in such conditions. For an unknown reason, most studies have focused on Westernized or China’s neighboring countries.

Recently, a team of authors proposed three new methods for studying the epidemiological course of COVID-19. The first one is a universal physics-based model designed to assess the COVID-19 dynamics in Europe. The model folds within the existing curve due to the fact that the results obtained following simulation indicate an evolution curve related to that describing the current status. This “overlap” can be explained by the fact that this approach is based on a universal mechanism, having as a structural concept, the “diffusion over a lattice”. In this context, it has been successfully applied for seven European countries, and further offers the chance to study the memory effects through autocorrelation within the epidemiological dynamical systems [22]. Furthermore, Demertzis et al. [23] applied an exploratory time-series analysis built on a recent conceptualization. More specific, is dedicated in detecting connective communities by developing a novel spline regression in which the knot vector is represented by the community detection in a complex network. Through this approach, the authors demonstrated the reliability of this exploratory time-series analysis in decision-making in Greece, mainly because diagnostic testing, services, and resources strategies vary between countries. Finally, Tsiotas et al. [24] used the modularity optimization algorithm in which the visibility graphs generated describe a sequence of different typologies that this disease has. According to their results, the current pandemic in Greece is about to reach the second half in a decreasing manner, whereas the chances for a “maximum infection” are low due to the saturation point reached.

Quarantine is the first alternative, Chintalapudi et al. [25] demonstrated that in Italy this approach promoted a reduction up to 35% of the total registered cases, in parallel with a significant percentage (66%) of recovered cases.

Considering the emphatic nature of humankind, self-isolation or quarantine could have branched and serious repercussions upon humans’ psychological profile. The psycho-social impact is exponential, post-traumatic stress disorder (PTSD) and depression representing just two examples [26]. The gut–brain axis (GBA) component should not be neglected, since it is already known that a long-term loss of host eubiosis can promote psychiatric or neurodegenerative disorders [27].

Based on the above discussed, from our point of view, a two-sided approach is social confinement. López et al. [28] considered that social confinement should remain valid for at least 8 weeks because 99% of the current wave was attributed to humans intervention and recommended a resumption of daily activities up to 50%. Chakraborty et al. [29] sustained the arguments of López taking into consideration that people >65 years are more prone, and consider the necessity of an adequate medical center arrangement.

A study conducted by Williamson et al. [30] in which reunited a cohort consisting of over 17 million UK people demonstrated an increased risk among Black and South Asian people, predisposition attributed to age, sex, and related medical conditions. Miller et al. [31] assumed a case scenario in which around 20% of the US population will be infected, especially counties compared to the rest of the country. The authors created this pattern based on a series of assumptions such as transmission, contact patterns, basic reproductive rate, and how efficient quarantine really is.

Despite that travel restriction and social distancing significantly reduce the risk of transmissibility, evidence regarding the use of face masks are inconsistent. Regardless of the status of the individual, even for an asymptomatic carrier, face masks can mitigate the risk [32]. A recent systematic review and meta-analysis conducted by Chu et al., [33] reunited 172 observations studies across 16 countries with a cohort consisting of 25,697 patients. As expected, the greater the physical distance than 1 m, the risk is inversely proportional and vice versa. Intriguingly, even eye protection was positively associated with less infection.

However, a question arises. Why is there such a significant difference in the total number of deaths between countries? A cross-sectional dataset comprising 169 countries aiming to investigate factors associated with cross-country variation revealed that mortality rate is influenced by a series of variables; government effectiveness, the number of hospital beds, transport infrastructure, and the most important is the number of tests performed [34].

If all these amendments will not be taken seriously, we could face a second wave much more severe [35], reflected by the number of deaths reported each day. A similar event has been recorded as a consequence of the violation of these prevention measures in Romania.

An investigation of 12,343 SARS-CoV-2 genome sequences coming from the individual from 6 distinct geographical regions revealed that ORF1ab 4715L and S protein 614G variants is in direct correlation with fatality rates. The authors also showed that the bacillus Calmette–Guérin (BCG) vaccine and the frequency of several HLA alleles are associated with fatality rates and the number of infected cases [36].

From our point of view, researchers and clinicians should change the direction of this topic. Where does the next question come from? “If it is still known that angiotensin-converting enzyme 2 (ACE2) receptors [37,38] are also found in different niches along the digestive tract, why is the number of studies that aim to identify SARS-CoV-2 using rectal swabs or stool samples limited?” In several previous occasions, it has been demonstrated the presence of viral signatures in stool samples starting from day seven, and ranging up to almost two weeks after infection [39,40,41,42]. This hypothesis is also supported by additional evidence that the incidence of gastrointestinal deficiencies varies from mild [40,43,44,45] to moderate [46,47,48,49].

The temperature could play an important role in the spreading of this virus. Demongeot et al. [50] concluded that high temperatures restrict the range of action of SARS-CoV-2, but this does not mean that in the cold season there will not be big question marks as to whether or not a person is infected with SARS-CoV-2, especially when it will overlap with influenza infections.

In conclusion, Eastern European countries such as Romania are at particular risk because of the vulnerabilities in the health system, corruption, and emigration of doctors. All these delays and the poor organization represent the consequences of the communist regime that still makes its mark even after more than three decades. It should be noted that Romania has also faced several economic crises, the critical point being reached on February 5 this year, at which point it collapsed [51].

Identical to Western models, and consistent with WHO guidelines (distance between people of about 1.5 m, wearing a mask, isolation, and massive testing), all these measures have been implemented also in Romania. Despite the efforts made, the sums allocated for carrying out such tests are insignificant, the equipment is missing, the staff is not qualified, and the hospitals are at full capacity.

Cumulatively, all these negative aspects are certified by an increasing number of infected people in contrast to the rest of Europe where the situation has reached the upper limit and is now stabilizing. What is certain is that Romania does not yet have an effective strategy to reduce the number of patients.

## 5. Conclusions

Forecasting the prevalence of SARS-CoV-2 is imperative to date, especially for health departments. As has been described and demonstrated throughout this study, time-series models play a crucial role in disease prediction. In this study, ARIMA time-series models were applied with success with the aim of estimating the overall prevalence of COVID-19 in Romania. However, based on our expertise and although both software have proven effective, Statgraphics has a much wider spectrum of possibilities in terms of speed, analysis, and utility. To these arguments is added the current pandemic, where providing a clear perspective in a short interval is vital for every individual.

## Figures and Tables

**Table 1:** Chronological presentation of various studies in which the ARIMA (AutoRegressive Integrated Moving Average) model was used.

Year of Publication	Disease	Method	Reference
2005	Severe Acute Respiratory Syndrome	ARIMA	[8]
2009	Malaria	ARIMA	[9]
2011	Hemorrhagic Fever with Renal Syndrome	ARIMA	[10]
2013	Hantavirus Pulmonary Syndrome	ARIMA	[11]
2015	Tuberculosis	ARIMA	[12]
2018	Influenza	ARIMA	[13]
2020	Brucellosis	ARIMA	[14]

**Table 2:** Descriptive statistics on the prevalence (**a**) and incidence (**b**) of COVID-19 in Romania.

**(a) Prevalence**							
**Interval**	**Mean**	**SE Mean**	**St. Dev**	**Minimum**	**Maximum**	**Skewness**	**Kurtosis**
1 March–31 March	482.866	118.415	648.588	3	2245	1.49	1.21
1 April–30 April	7603.551	540.324	2909.737	2738	12,240	−0.03	−1.23
1 May–31 May	16,502.933	346.883	1899.957	12,732	19,257	−0.37	−0.90
1 June–30 June	22,776.689	427.610	2302.754	19,517	26,970	0.31	−1.16
1 July–31 July	36,874.3	1287.582	7052.379	27,746	50,886	0.61	−0.94
1 August–31 August	70,602.366	1930.889	10,575.917	53,186	87,540	−0.03	−1.19
1 March–31 August	25,840.071	1751.969	23,700.201	3	87,540	1.04	0.17
**(b) Incidence**							
**Interval**	**Mean**	**SE Mean**	**St. Dev**	**Minimum**	**Maximum**	**Skewness**	**Kurtosis**
1 March–31 March	74.733	17.058	93.434	0	308	1.33	0.69
1 April–30 April	337.241	16.016	86.250	190	523	0.31	−0.38
1 May–31 May	223	14.369	78.704	124	431	1.08	0.59
1 June–30 June	261.103	16.507	88.896	119	460	0.40	−0.39
1 July–31 July	786.333	59.450	325.623	250	1356	0.19	−1.28
1 August–31 August	1180.966	42.478	232.664	733	1504	−0.63	−0.85
1 March–31 August	478.344	31.352	424.128	0	1504	1.03	−0.24

**Table 3:** Comparison of tested ARIMA models.

**(a) STATGRAPHICS Centurion (v.18.1.13)**				
**Romania**	**Model**	**RMSE**	**MAE**	**MAPE**
March	(1,2,1)	40.2064	21.7726	9.3225
	(2,2,0)	40.1344	21.8332	9.33149
	(2,1,0)	37.1349	22.3392	9.42158
	(3,2,0)	40.7137	22.252	9.50679
	(3,0,0)	36.317	21.4381	9.58606
April	(3,2,2)	84.4845	62.4813	0.975287
	(3,2,3)	91.4283	64.3356	0.978607
	(3,2,1)	86.0235	63.5418	0.988232
	(1,2,3)	86.1254	66.3094	1.03015
	(0,2,3)	84.8321	66.818	1.03804
May	(3,1,3)	55.1218	35.4972	0.227675
	(3,2,3)	51.5543	37.7316	0.233695
	(3,2,2)	52.2601	37.5565	0.235246
	(3,2,1)	52.0651	37.7596	0.235816
	(3,2,0)	51.9334	38.9065	0.243301
June	(3,2,2)	53.3883	36.8425	0.161412
	(3,2,3)	55.042	36.8814	0.161561
	(3,1,3)	66.319	45.2191	0.195068
	(2,1,3)	66.8927	47.8626	0.207124
July	(3,1,3)	117.982	87.1512	0.243285
	(2,1,1)	113.198	88.1797	0.24369
	(2,1,2)	115.679	88.7863	0.245774
	(1,1,2)	115.307	92.5001	0.256055
August	(2,2,2)	153.804	113.314	0.163873
	(3,2,2)	155.701	114.742	0.164574
	(3,2,3)	159.39	115.195	0.165348
March–August	(1,2,1)	121.674	85.2619	2.29175
	(3,2,3)	118.411	82.2194	2.37771
	(1,2,3)	118.36	82.5649	2.37918
	(3,2,1)	113.778	80.2205	2.40063
	(3,2,0)	121.301	84.6413	2.41403
**(b) IBM SPSS (v.20.0.0)**				
**Romania**	**Model**	**RMSE**	**MAE**	**MAPE**
March	(1,2,1)	38.127	24.651	57.505
April	(3,2,2)	96.089	68.365	1.152
May	(3,1,3)	68.403	39.996	0.259
June	(3,2,2)	58.588	41.854	0.185
July	(3,1,3)	156.476	106.572	0.307
August	(2,2,2)	179.309	129.350	0.194
March–August	(1,2,1)	121.054	85.524	6.013

**Table 4:** Parameters of ARIMA models.

**(a) STATGRAPHICS Centurion (v.18.1.13)**						
**Romania**	**Parameters**	**Estimate**	**Standard Error**		***t*-Statistic**	***p*-Value**
March (1,2,1)	AR(1) MA(1)	−0.865514 −0.209212	0.194131 0.291261		−4.45841 −0.718298	0.000131 0.478744
April (3,2,2)	AR(3) MA(2)	−0.329312 −0.528086	0.225307 0.247375		−1.46161 −2.13475	0.157377 0.043660
May (3,1,3)	AR(3) MA(3)	0.625887 0.570657	0.145922 0.0544548		4.28918 10.4795	0.000253 0.000000
June (3,2,2)	AR(3) MA(2)	−0.312216 −0.964198	0.209998 0.0269271		−1.48676 −35.8077	0.150660 0.000000
July (3,1,3)	AR(3) MA(3)	−0.560219 −0.0478648	0.258752 0.274587		−2.16508 −0.174315	0.040545 0.863080
August (2,2,2)	AR(2) MA(2)	−0.826566 −0.782937	0.112664 0.171731		−7.33655 −4.5591	0.000000 0.000117
March–August (1,2,1)	AR(1) MA(1)	0.479999 0.781228	0.122096 0.0765947		3.93133 10.1995	0.000120 0.000000
**(b) IBM SPSS (v.20.0.0)**						
**ARIMA Model Parameters**						
					Estimate	Standard Error
Cumulative-Model (March)	Cumulative	No Transformation	Constant		8.881	3.986
			AR	Lag 1	−0.740	0.206
			Difference		2	
			MA	Lag 1	0.037	0.287
					*t*-statistic	*p*-value
Cumulative-Model (March)	Cumulative	No Transformation	Constant		2.228	0.035
			AR	Lag 1	−3.595	−0.001
			Difference			
			MA	Lag 1	0.130	0.898
					Estimate	Standard Error
Cumulative-Model (April)	Cumulative	No Transformation	Constant		−0.902	1.468
			AR	Lag 1	0.210	0.486
				Lag 2	−0.216	0.203
				Lag 3	−0.268	0.257
			Difference		2	
			MA	Lag 1	1.527	22.293
				Lag 2	−0.528	11.600
					*t*-statistic	*p*-value
Cumulative-Model (April)	Cumulative	No Transformation	Constant		−0.615	0.545
			AR	Lag 1	0.432	0.670
				Lag 2	−1.065	0.298
				Lag 3	−1.042	0.309
			Difference			
			MA	Lag 1	0.069	0.946
				Lag 2	−0.046	0.964
					Estimate	Standard Error
Cumulative-Model (May)	Cumulative	No Transformation	Constant		216.917	46.423
			AR	Lag 1	−0.013	0.498
				Lag 2	0.252	0.348
				Lag 3	0.480	0.348
			Difference		1	
			MA	Lag 1	−0.641	3.924
				Lag 2	−0.258	4.043
				Lag 3	0.591	3.576
					*t*-statistic	*p*-value
Cumulative-Model (May)	Cumulative	No Transformation	Constant		4.673	0.000
			AR	Lag 1	−0.026	0.980
				Lag 2	0.725	0.476
				Lag 3	1.381	0.181
			Difference			
			MA	Lag 1	−0.163	0.872
				Lag 2	−0.064	0.950
				Lag 3	0.165	0.870
					Estimate	Standard Error
Cumulative-Model (June)	Cumulative	No Transformation	Constant		8.194	1.482
			AR	Lag 1	0.161	0.487
				Lag 2	−0.159	0.291
				Lag 3	−0.451	0.234
			Difference		2	
			MA	Lag 1	0.914	4.922
				Lag 2	0.080	0.849
					*t-*statistic	*p*-value
Cumulative-Model (June)	Cumulative	No Transformation	Constant		5.529	0.000
			AR	Lag 1	0.332	0.743
				Lag 2	−0.549	0.589
				Lag 3	−1.928	0.067
			Difference			
			MA	Lag 1	0.186	0.854
				Lag 2	0.094	0.926
					Estimate	Standard Error
Cumulative-Model (July)	Cumulative	No Transformation	Constant		837.899	505.059
			AR	Lag 1	0.274	20.176
				Lag 2	0.753	3.824
				Lag 3	−0.087	15.011
			Difference		1	
			MA	Lag 1	−0.629	20.192
				Lag 2	0.259	14.442
				Lag 3	−0.003	3.383
					*t*-statistic	*p*-value
Cumulative-Model (July)	Cumulative	No Transformation	Constant		1.659	0.111
			AR	Lag 1	0.014	0.989
				Lag 2	0.197	0.846
				Lag 3	−0.006	0.995
			Difference			
			MA	Lag 1	−0.031	0.975
				Lag 2	0.018	0.986
				Lag 3	−0.001	0.999
					Estimate	Standard Error
Cumulative-Model (August)	Cumulative	No Transformation	Constant		−4.351	1.253
			AR	Lag 1	1.120	0.139
				Lag 2	−0.832	0.107
			Difference		2	
			MA	Lag 1	1.978	6.107
				Lag 2	−0.995	6.084
					*t*-statistic	*p*-value
Cumulative-Model (August)	Cumulative	No Transformation	Constant		−3.474	0.002
			AR	Lag 1	8.077	0.000
				Lag 2	−7.804	0.000
			Difference			
			MA	Lag 1	0.324	0.749
				Lag 2	−0.164	0.871
					Estimate	Standard Error
Cumulative-Model (March–August)	Cumulative	No Transformation	Constant		5.885	3.318
			AR	Lag 1	0.501	0.126
			Difference		2	
			MA	Lag 1	0.820	0.084
					*t*-statistic	*p*-value
Cumulative-Model (March–August)	Cumulative	No Transformation	Constant		1.773	0.078
			AR	Lag 1	3.985	0.000
			Difference			
			MA	Lag 1	9.712	0.000

**Table 5:** Prediction of total confirmed cases of COVID−19 for the next two weeks through (**a**) STATGRAPHICS Centurion (v.18.1.13) and (**b**) IBM SPSS (v.20.0.0).

**(a) STATGRAPHICS Centurion (v.18.1.13)**							
**March (1,2,1)**							
				*Lower 95%*		*Upper 95%*	
*Period*	*Forecast*			*Limit*		*Limit*	
01-4-20	2450.74			2368.24		2533.23	
02-4-20	2732.0			2593.82		2870.18	
03-4-20	2947.89			2716.13		3179.66	
04-4-20	3220.37			2900.32		3540.42	
05-4-20	3443.87			3011.65		3876.09	
06-4-20	3709.76			3166.05		4253.47	
07-4-20	3938.96			3266.6		4611.32	
08-4-20	4199.91			3397.23		5002.6	
09-4-20	4433.39			3487.15		5379.63	
10-4-20	4690.65			3597.86		5783.43	
11-4-20	4927.32			3677.29		6177.34	
12-4-20	5181.81			3770.81		6592.81	
13-4-20	5420.88			3839.91		7001.84	
14-4-20	5673.29			3918.22		7428.36	
**April (3,2,2)**							
				*Lower 95%*		*Upper 95%*	
*Period*	*Forecast*			*Limit*		*Limit*	
01-5-20	12,616.5			12,440.4		12,792.5	
02-5-20	12,959.9			12,738.5		13,181.4	
03-5-20	13,302.9			13,061.4		13,544.4	
04-5-20	13,615.5			13,368.8		13,862.3	
05-5-20	13,932.3			13,674.7		14,189.9	
06-5-20	14,257.0			13,975.5		14,538.5	
07-5-20	14,592.6			14,276.6		14,908.7	
08-5-20	14,927.5			14,579.7		15,275.3	
09-5-20	15,257.2			14,883.3		15,631.0	
10-5-20	15,582.2			15,184.3		15,980.1	
11-5-20	15,907.6			15,482.8		16,332.4	
12-5-20	16,235.9			15,779.8		16,692.0	
13-5-20	16,566.2			16,076.4		17,056.0	
14-5-20	16,896.3			16,372.8		17,419.8	
**May (3,1,3)**							
				*Lower 95%*		*Upper 95%*	
*Period*	*Forecast*			*Limit*		*Limit*	
01-6-20	19,400.9			19,280.2		19,521.5	
02-6-20	19,533.0			19,286.3		19,779.8	
03-6-20	19,689.2			19,296.5		20,081.9	
04-6-20	19,831.8			19,307.7		20,355.9	
05-6-20	19,971.4			19,290.8		20,651.9	
06-6-20	20,122.2			19,270.7		20,973.7	
07-6-20	20,265.2			19,240.9		21,289.5	
08-6-20	20,408.1			19,194.8		21,621.5	
09-6-20	20,556.2			19,143.4		21,968.9	
10-6-20	20,699.9			19,081.8		22,318.0	
11-6-20	20,844.3			19,009.0		22,679.7	
12-6-20	20,991.0			18,929.9		23,052.1	
13-6-20	21,135.4			18,841.3		23,429.4	
14-6-20	21,280.5			18,744.1		23,816.9	
**June (3,2,2)**							
				*Lower 95%*		*Upper 95%*	
*Period*	*Forecast*			*Limit*		*Limit*	
01-7-20	27,404.9			27,291.5		27,518.3	
02-7-20	27,860.4			27,673.0		28,047.7	
03-7-20	28,267.6			28,019.4		28,515.8	
04-7-20	28,608.4			28,307.9		28,908.9	
05-7-20	28,916.8			28,551.9		29,281.8	
06-7-20	29,253.3			28,792.0		29,714.6	
07-7-20	29,652.8			29,060.9		30,244.7	
08-7-20	30,097.5			29,360.4		30,834.6	
09-7-20	30,533.1			29,658.3		31,407.9	
10-7-20	30,914.6			29,916.3		31,912.9	
11-7-20	31,243.0			30,126.4		32,359.6	
12-7-20	31,562.8			30,317.0		32,808.5	
13-7-20	31,924.0			30,526.8		33,321.1	
14-7-20	32,340.9			30,772.3		33,909.5	
**July (3,1,3)**							
				*Lower 95%*		*Upper 95%*	
*Period*	*Forecast*			*Limit*		*Limit*	
01-8-20	52,247.6			51,999.2		52,496.1	
02-8-20	53,668.7			53,169.6		54,167.9	
03-8-20	55,147.3			54,390.9		55,903.6	
04-8-20	56,685.2			55,656.5		57,714.0	
05-8-20	58,282.6			56,976.8		59,588.5	
06-8-20	59,942.3			58,346.7		61,537.9	
07-8-20	61,665.3			59,772.1		63,558.4	
08-8-20	63,454.6			61,250.0		65,659.3	
09-8-20	65,311.9			62,784.7		67,839.2	
10-8-20	67,240.4			64,374.9		70,106.0	
11-8-20	69,242.1			66,024.5		72,459.8	
12-8-20	71,320.4			67,733.2		74,907.6	
13-8-20	73,477.6			69,504.9		77,450.3	
14-8-20	75,717.2			71,340.0		80,094.4	
**August (2,2,2)**							
				*Lower 95%*		*Upper 95%*	
*Period*	*Forecast*			*Limit*		*Limit*	
01-9-20	88,483.4			88,163.3		88,803.4	
02-9-20	89,735.8			89,186.2		90,285.4	
03-9-20	91,171.6			90,521.1		91,822.0	
04-9-20	92,553.1			91,883.1		93,223.0	
05-9-20	93,723.4			93,050.4		94,396.4	
06-9-20	94,707.0			94,020.1		95,393.9	
07-9-20	95,660.3			94,899.8		96,420.7	
08-9-20	96,734.6			95,825.9		97,643.3	
09-9-20	97,966.8			96,901.4		99,032.2	
10-9-20	99,272.2			98,093.1		100,451.	
11-9-20	100,527.			99,273.2		101,781.	
12-9-20	101,667.			100,347.		102,986.	
13-9-20	102,721.			101,316.		104,126.	
14-9-20	103,777.			102,249.		105,305.	
**March–August (1,2,1)**							
				*Lower 95%*		*Upper 95%*	
*Period*	*Forecast*			*Limit*		*Limit*	
01-9-20	88,427.4			88,187.3		88,667.5	
02-9-20	89,378.4			88,905.1		89,851.7	
03-9-20	90,359.9			89,641.2		91,078.7	
04-9-20	91,356.1			90,382.1		92,330.1	
05-9-20	92,359.3			91,120.4		93,598.2	
06-9-20	93,365.8			91,851.8		94,879.9	
07-9-20	94,374.0			92,574.3		96,173.7	
08-9-20	95,383.0			93,286.8		97,479.2	
09-9-20	96,392.3			93,988.7		98,796.0	
10-9-20	97,401.8			94,679.8		100,124.	
11-9-20	98,411.4			95,360.3		101,463.	
12-9-20	99,421.1			96,030.1		102,812.	
13-9-20	100,431.			96,689.5		104,172.	
14-9-20	101,440.			97,338.6		105,542.	
**(b) IBM SPSS (v.20.0.0)**							
**Forecast**							
Model		**Day 1**	**Day 2**	**Day 3**	**Day 4**	**Day 5**	**Day 6**
Cumulative-Model (March)	Forecast	2478.78	2771.81	3036.46	3337.56	3627.14	3940.69
	UCL	2557.11	2895.56	3237.39	3612.37	3992.87	4399.42
	LCL	2400.44	2648.06	2835.53	3062.74	3261.42	3481.96
Model		**Day 7**	**Day 8**	**Day 9**	**Day 10**	**Day 11**	**Day 12**
Cumulative-Model (March)	Forecast	4251.96	4580.37	4911.55	5256.13	5606.25	5967.72
	UCL	4814.73	5251.20	5698.58	6164.03	6641.61	7135.33
	LCL	3689.20	3909.55	4124.53	4348.24	4570.89	4800.11
Model		**Day 13**			**Day 14**		
Cumulative-Model (March)	Forecast	6336.24			6715.00		
	UCL	7641.78			8163.17		
	LCL	5030.71			5266.84		
Forecast							
Model		**Day 1**	**Day 2**	**Day 3**	**Day 4**	**Day 5**	**Day 6**
Cumulative-Model (April)	Forecast	12,599.76	12,935.87	13,271.55	13,584.87	13,898.80	14,216.66
	UCL	12,774.44	13,150.93	13,500.98	13,816.96	14,136.69	14,467.65
	LCL	12,425.08	12,720.81	13,042.12	13,352.78	13,660.91	13,965.67
Model		**Day 7**	**Day 8**	**Day 9**	**Day 10**	**Day 11**	**Day 12**
Cumulative-Model (April)	Forecast	14,540.05	14,862.45	15,181.23	15,496.84	15,811.68	16,126.86
	UCL	14,808.71	15,145.84	15,475.55	15,800.64	16,125.74	16,452.34
	LCL	14,271.39	14,579.05	14,886.92	15,193.03	15,497.61	15,801.38
Model		**Day 13**			**Day 14**		
Cumulative-Model (April)	Forecast	16,441.99			16,756.08		
	UCL	16,779.10			17,104.18		
	LCL	16,104.88			16,407.98		
Forecast							
Model		**Day 1**	**Day 2**	**Day 3**	**Day 4**	**Day 5**	**Day 6**
Cumulative-Model (May)	Forecast	19,412.18	19,569.68	19,763.17	19,935.78	20,118.83	20,313.78
	UCL	19,543.30	19,816.82	20,130.38	20,397.32	20,688.05	20,989.84
	LCL	19,281.05	19,322.54	19,395.96	19,474.23	19,549.62	19,637.71
Model		**Day 7**	**Day 8**	**Day 9**	**Day 10**	**Day 11**	**Day 12**
Cumulative-Model (May)	Forecast	20,501.17	20,696.67	20,895.88	21,093.45	21,295.88	21,499.60
	UCL	21,277.17	21,576.47	21,877.30	22,173.28	22,473.47	22,773.08
	LCL	19,725.17	19,816.88	19,914.45	20,013.63	20,118.28	20,226.12
Model		**Day 13**			**Day 14**		
Cumulative-Model (May)	Forecast	21,703.75			21,910.55		
	UCL	23,071.27			23,369.65		
	LCL	20,336.23			20,451.44		
Forecast							
Model		**Day 1**	**Day 2**	**Day 3**	**Day 4**	**Day 5**	**Day 6**
Cumulative-Model (June)	Forecast	27,405.69	27,851.25	28,248.97	28,627.75	29,018.52	29,447.71
	UCL	27,520.95	28,038.23	28,481.50	28,874.60	29,273.56	29,714.32
	LCL	27,290.42	27,664.28	28,016.44	28,380.90	28,763.48	29,181.10
Model		**Day 7**	**Day 8**	**Day 9**	**Day 10**	**Day 11**	**Day 12**
Cumulative-Model (June)	Forecast	29,901.63	30,359.87	30,809.40	31,257.55	31,716.79	32,193.86
	UCL	30,190.48	30,674.74	31,145.84	31,609.14	32,081.24	32,572.50
	LCL	29,612.78	30,045.00	30,472.95	30,905.96	31,352.35	31,815.21
Model		**Day 13**			**Day 14**		
Cumulative-Model (June)	Forecast	32,684.53			33,181.42		
	UCL	33,079.98			33,594.43		
	LCL	32,289.08			32,768.41		
Forecast							
Model		**Day 1**	**Day 2**	**Day 3**	**Day 4**	**Day 5**	**Day 6**
Cumulative-Model (July)	Forecast	52,168.29	53,426.67	54,674.30	55,902.11	57,118.49	58,317.76
	UCL	52,449.73	54,031.51	55,633.07	57,264.85	58,911.15	60,573.52
	LCL	51,886.84	52,821.82	53,715.54	54,539.37	55,325.84	56,061.99
Model		**Day 7**	**Day 8**	**Day 9**	**Day 10**	**Day 11**	**Day 12**
Cumulative-Model (July)	Forecast	59,505.46	60,678.11	61,839.43	62,987.33	64,124.34	65,249.25
	UCL	62,244.36	63,923.34	65,606.40	67,292.68	68,979.84	70,666.96
	LCL	56,766.56	57,432.88	58,072.46	58,681.98	59,268.84	59,831.54
Model		**Day 13**			**Day 14**		
Cumulative-Model (July)	Forecast	66,363.83			67,467.42		
	UCL	72,352.60			74,035.96		
	LCL	60,375.05			60,898.88		
Forecast							
Model		**Day 1**	**Day 2**	**Day 3**	**Day 4**	**Day 5**	**Day 6**
Cumulative-Model (August)	Forecast	88,444.38	89,634.01	91,015.67	92,372.04	93,537.29	94,506.51
	UCL	88,730.43	90,079.75	91,493.00	92,852.85	94,048.38	95,027.52
	LCL	88,158.32	89,188.27	90,538.35	91,891.23	93,026.19	93,985.50
Model		**Day 7**	**Day 8**	**Day 9**	**Day 10**	**Day 11**	**Day 12**
Cumulative-Model (August)	Forecast	95,412.10	96,406.37	97,549.71	98,783.13	99,990.33	101,090.16
	UCL	95,938.80	96,978.64	98,171.45	99,421.94	100,629.11	101,728.19
	LCL	94,885.41	95,834.09	96,927.97	98,144.32	99,351.54	100,452.13
Model		**Day 13**			**Day 14**		
Cumulative-Model (August)	Forecast	102,088.51			103,059.41		
	UCL	102,726.16			103,708.74		
	LCL	101,450.85			102,410.08		
Forecast							
Model		**Day 1**	**Day 2**	**Day 3**	**Day 4**	**Day 5**	**Day 6**
Cumulative-Model (March–August)	Forecast	88,451.83	89,445.12	90,482.12	91,543.96	92,621.15	93,708.98
	UCL	88,690.71	89,912.30	91,185.57	92,489.10	93,813.54	95,154.94
	LCL	88,212.96	88,977.94	89,778.67	90,598.81	91,428.77	92,263.03
Model		**Day 7**	**Day 8**	**Day 9**	**Day 10**	**Day 11**	**Day 12**
Cumulative-Model (March–August)	Forecast	94,805.08	95,908.24	97,017.89	98,133.72	99,255.59	100,383.41
	UCL	96,511.68	97,883.14	99,269.09	100,669.45	102,084.15	103,513.14
	LCL	93,098.47	93,933.34	94,766.69	95,598.00	96,427.02	97,253.68
Model		**Day 13**			**Day 14**		
Cumulative-Model (March–August)	Forecast	101,517.16			102,656.81		
	UCL	104,956.35			106,413.66		
	LCL	98,077.97			98,899.95		
